# *TP53* and Let-7a micro-RNA Regulate K-Ras Activity in HCT116 Colorectal Cancer Cells

**DOI:** 10.1371/journal.pone.0070604

**Published:** 2013-08-01

**Authors:** Carrie Luu, Eileen L. Heinrich, Marjun Duldulao, Amanda K. Arrington, Marwan Fakih, Julio Garcia-Aguilar, Joseph Kim

**Affiliations:** 1 Division of Surgical Oncology, Department of Surgery, City of Hope Comprehensive Cancer Center, Duarte, California, United States of America; 2 Department of Medical Oncology and Experimental Therapeutics, City of Hope Comprehensive Cancer Center, Duarte, California, United States of America; 3 Department of Surgery, Memorial Sloan-Kettering Cancer Center, New York, New York, United States of America; Consiglio Nazionale delle Ricerche (CNR), Italy

## Abstract

Recent reports have indicated that *KRAS* and *TP53* mutations predict response to therapy in colorectal cancer. However, little is known about the relationship between these two common genetic alterations. Micro-RNAs (miRNAs), a class of noncoding RNA implicated in cellular processes, have been increasingly linked to *KRAS* and *TP53*. We hypothesized that lethal-7a (let-7a) miRNA regulates *KRAS* through *TP53*. To investigate the relationship between *KRAS, TP53*, and let-7a, we used HCT116 *KRAS^mut^* human colorectal cancer cells with four different genotypic modifications in *TP53* (*TP53^−/−^, TP53^+/−^, TP53^mut/+^,* and *TP53^mut/−^*). Using these cells we observed that K-Ras activity was higher in cells with mutant or knocked out *TP53* alleles, suggesting that wild-type *TP53* may suppress K-Ras activity. Let-7a was present in HCT116 *KRAS^mut^* cells, though there was no correlation between let-7a level and *TP53* genotype status. To explore how let-7a may regulate K-Ras in the different *TP53* genotype cells we used let-7a inhibitor and demonstrated increased K-Ras activity across all *TP53*, thus corroborating prior reports that let-7a regulates K-Ras. To assess potential clinical implications of this regulatory network, we examined the influence of *TP53* genotype and let-7a inhibition on colon cancer cell survival following chemoradiation therapy (CRT). We observed that cells with complete loss of wild-type *TP53* alleles (^−/−^ or ^−/mut^) were resistant to CRT following treatment with 5-fluorouracil and radiation. Further increase in K-Ras activity with let-7a inhibition did not impact survival in these cells. In contrast, cells with single or double wild-type *TP53* alleles were moderately responsive to CRT and exhibited resistance when let-7a was inhibited. In summary, our results show a complex regulatory system involving *TP53*, *KRAS*, and let-7a. Our results may provide clues to understand and target these interactions in colorectal cancer.

## Introduction

Colorectal cancer (CRC) is the 4^th^ most common cancer and the 2^nd^ most common cause of cancer-related death in the US [1]. Recent advances that have improved outcomes in CRC have included the identification of accurate prognostic and predictive molecular biomarkers [2]–[4]. These have included genetic alterations in *KRAS* and *TP53*, which are frequently detected in patients with CRC. *KRAS* mutations are present in approximately 30–50% of CRCs, but also in 17–25% of all human tumors [2], [5]–[7]. Similarly, *TP53* alterations are common in patients with CRC (nearly 50%) [8]. Both prognostic and predictive roles have been identified for both genes [9]. Our own group and others has recently shown that detection of concurrent *KRAS* and *TP53* mutations, with an incidence of 5% to 20% in CRC patients, correlated with resistance to neoadjuvant chemoradiation therapy (CRT) in patients with rectal cancer [10]–[12]. Despite the frequency of these mutations in CRC, little is known about interactions between the two genes.

The link between these two frequently altered genes in CRC may lie in micro-RNA (miRNA), a class of non-coding RNA which function in transcriptional regulation and more specifically may influence the regulation of cell proliferation and apoptosis [13], [14]. Recent reports have suggested that the tumor suppressor activity of miRNA lethal 7a (let-7a) may be due to its association with *KRAS* and that inhibition of tumor growth may occur by suppression of K-Ras expression by let-7a [15], [16]. Emerging clinical data suggest that intra-tumor let-7a expression correlates with tumor response and overall survival in metastatic colorectal cancer patients receiving epidermal growth factor (EGFR) targeting agents in both *KRAS* wild-type and mutant populations [17]. Additional studies have speculated that the role of *TP53* in DNA repair and apoptosis may in part be regulated by miRNAs, including let-7a [18], [19]. Therefore, a complex regulatory network for *TP53* and *KRAS* may be linked by let-7a.

To investigate potential interactions between *TP53*, *KRAS*, and let-7a, we analyzed a unique family of colorectal cancer cell lines with mutant *KRAS* and modifications in *TP53* genotype. These cells enabled us to examine the changes in K-Ras expression and activity that corresponded with variations in *TP53* genotype. Furthermore, we were able to better interrogate the role of let-7a in the setting of mutant *KRAS* and various *TP53* genotypes. Our results indicate novel increases in K-Ras activity with different *TP53* genotypes. However, we did not find a clear relationship between let-7a level and *TP53* genotype. Nonetheless, changes in K-Ras activity were regulated by let-7a. This first report of *TP53* and let-7a regulation of K-Ras activity provides clues to better understand the complex interaction between *TP53* and *KRAS*.

## Materials and Methods

### Cell Culture

The human CRC cell line HCT116, harboring a single mutant *KRAS* allele and double wild-type *TP53* (*TP53^+/+^*) alleles, was modified into four stable cell lines with different *TP53* genotypes (*TP53^−/−^, TP53^+/−^, TP53^mut/+^,* and *TP53^mut/−^*). The parent and modified cell lines were kindly provided by Dr. Bert Vogelstein (Johns Hopkins University; Baltimore, MD) [20]. Mutant and knockout alleles were both used to assess potential differences between the two alleles, as suggested in prior reports [21]. All cell lines were assessed by DNA extraction, polymerase chain reaction (PCR), and sequencing for *KRAS* and *TP53* gene mutations to verify genotypes. Cells were maintained in McCoy 5A medium (Irvine Scientific; Santa Ana, CA) with 10% fetal bovine serum (Omega Scientific; Tarzana, CA) and 1% penicillin-streptomycin-glutamine (Invitrogen; Carlsbad, CA) at 5% CO_2_ at 37°C.

### Immunoblotting

Cell lysates were collected using RIPA buffer (Invitrogen; Carlsbad, CA) plus protease inhibitors (Thermo Scientific; Rockford, IL). Twenty micrograms of protein were separated on 12% SDS-polyacrylamide gels and transferred onto PVDF membranes (Millipore; Bedford, MA). The membranes were blocked for 1 h with 5% non-fat dry milk and probed overnight with primary antibodies. After washing, membranes were labeled with horseradish peroxidase (HRP)-conjugated secondary antibodies (BioRad; Hercules, CA). Membranes were developed using a chemiluminescent substrate (Amersham Pharmacia; Piscataway, NJ) and imaged. Antibodies used were: anti-K-Ras (Millipore) and anti-GAPDH (Cell Signaling; Danvers, MA).

### K-Ras Activity

K-Ras activity was measured using the Ras Activation ELISA Assay Kit (Millipore). In brief, cell lysates were incubated with Raf-1 Ras Binding Domain (RBD)-agarose. Raf-1-RBD was used to capture the active GTP-bound K-Ras protein, which was then detected by the addition of an anti-K-Ras-antibody (Millipore). An HRP-conjugated secondary antibody was then added. A luminometer was used to measure the signals after addition of a chemiluminescent reagent (Perkin-Elmer; Shelton, CT). Since assays were performed to assess the relative changes in K-Ras activity among the different *TP53* genotypes, K-Ras activity from the parental *TP53* ^+/+^ line was set as 1. For assays with let-7a inhibitor, each genotype acted as its own control. Three independent assays were performed for each cell line, and the mean absorbance ± the standard deviation (SD) was plotted for each line.

### Reverse Transcription

RT-PCR was performed using the Taqman ® miRNA assay (Invitrogen) following the manufacturer’s protocol. Briefly, reverse transcription was performed using the provided Multiscribe RT™ Reagent master mix plus cell lysates and the let-7a specific primers (Invitrogen). Reactions were performed in a thermal cycler at 16°C for 30 min, 42°C for 30 min, and 85°C for 5 min.

### Real Time-PCR

RT-PCR was performed using the Taqman® miRNA Cells to CT™ Kit (Invitrogen) according to the manufacturer’s protocol. Briefly, the RT product was added to the PCR cocktail (Taqman® miRNA assay reagent plus provided Taqman® master mix) and the RT-PCR reactions were run at 95°C for 10 min, followed by 40 cycles of 15 sec at 95°C and 1 min at 60°C in the Applied Biosystems 7500 fast RT-PCR system (Foster City, CA). Each sample was assayed in triplicate.

### Let-7a Inhibitor

Let-7a was inhibited by miRIDIAN miRNA inhibitors (Dharmacon; Lafeyette, CO) following the manufacturer’s protocol. Briefly, HCT116 cells (3×10^5^) were plated in 6-well plates and incubated overnight. They were then transfected with 100 nM of miRIDIAN miRNA inhibitor for 24 h. Cells were incubated for an additional 48 h prior to being utilized in assays.

### Chemoradiation Therapy

HCT116 cells were treated with CRT according to an established protocol [22]. After a total of 72 h incubation with let-7a inhibitor, cells were plated in 6-well plates and incubated overnight. Cells were then treated with 4 nM 5-Fluorouracil (5-FU) (Sigma Aldrich; St. Louis, MO) for 16 h after which they were exposed to 4 Gy of radiation. Drug exposure was halted 6 h after radiation treatment by medium exchange. Cells that had not been treated with let-7a inhibitor were similarly treated with CRT.

### Determination of Cell Viability

Cell viability was assessed using an ATP-based assay (Cell Titer-Glo; Promega; Madison, WI) as per manufacturer’s protocol. Briefly, HCT116 cells (5×10^3^) were plated in 96-well plates and incubated overnight. Cell Titer-Glo reagent was added and cell lysis induced. Luminescence was recorded with a luminometer. Three independent experiments were performed. Data shown represent the average viability, as a percent of the viability of untreated cells.

### Statistical Analysis

Statistical analysis was performed on Microsoft excel software (Microsoft; Redmond, WA). Each data point represents the average value of three independent experiments. Control and treatment conditions were compared by Student’s t-test and differences among cell lines were compared by analysis of variance (ANOVA). P value <0.05 was considered statistically significant.

## Results

### K-Ras Protein Level is not Dependent on *TP53* Mutation Status

**Figure 1:**
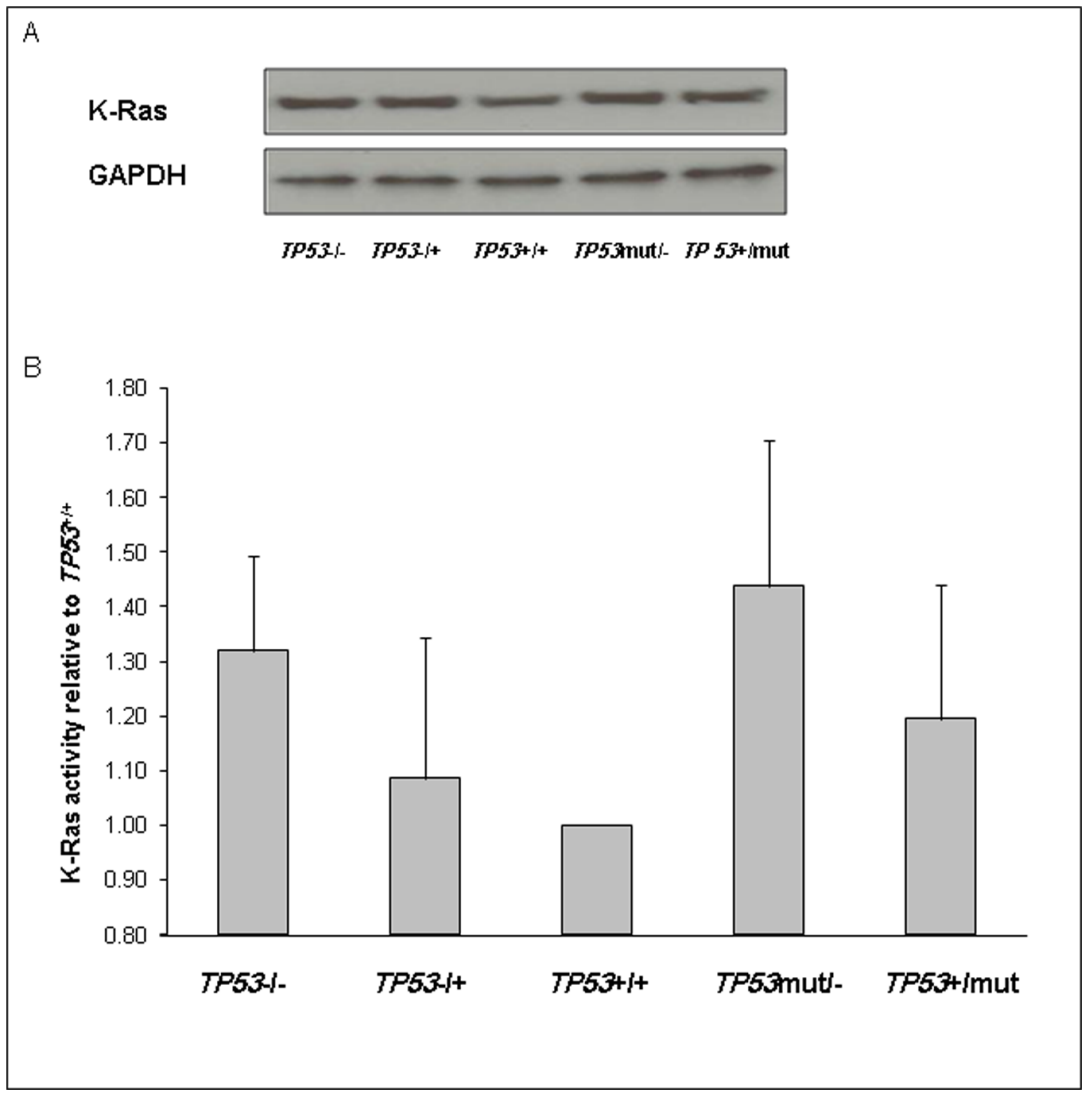
K-Ras expression is not dependent on *TP53* mutational status but K-Ras activity is increased in all cells compared to *TP53* ^+/+^ cells. A) HCT116 cells with the noted variations in *TP53* mutation status were collected and lysed for western blotting for K-Ras expression. GAPDH was used as a loading control. B) K-Ras activity was measured in all cell lines using a Raf pull-down ELISA assay. Results shown represent the average of 3 independent experiments performed in triplicate relative to *TP53*+/+ cells, p<0.05. Error bars show standard deviation (SD).

To determine the effect of different *TP53* genotypes on K-Ras expression, western blot assay was performed to compare protein levels. Our results show that K-Ras expression levels were not influenced by the different *TP53* genotypes (Figure 1A) suggesting that *TP53* does not regulate K-Ras protein expression.

### Effect of *TP53* Mutation Status on K-Ras Activity

The effect of *TP53* genotype on K-Ras activity was measured using a Raf pull-down assay. We discovered that K-Ras activity was lowest in *TP53^+/+^* cells. Increased K-Ras activity was most pronounced in *TP53* ^mut/−^, followed by *TP53^−/−^,* TP53^+/mut^, and TP53^−/+^ (44%, 32%, 20%, and 10% increases, respectively, p<0.05) (Figure 1B). These results show that K-Ras activity was lowest in cells with wild-type *TP53* alleles; and the highest levels were identified in cells with homozygous loss of the wild-type TP53 alleles.

### Let-7a is Expressed in All HCT116 Lines

**Figure 2:**
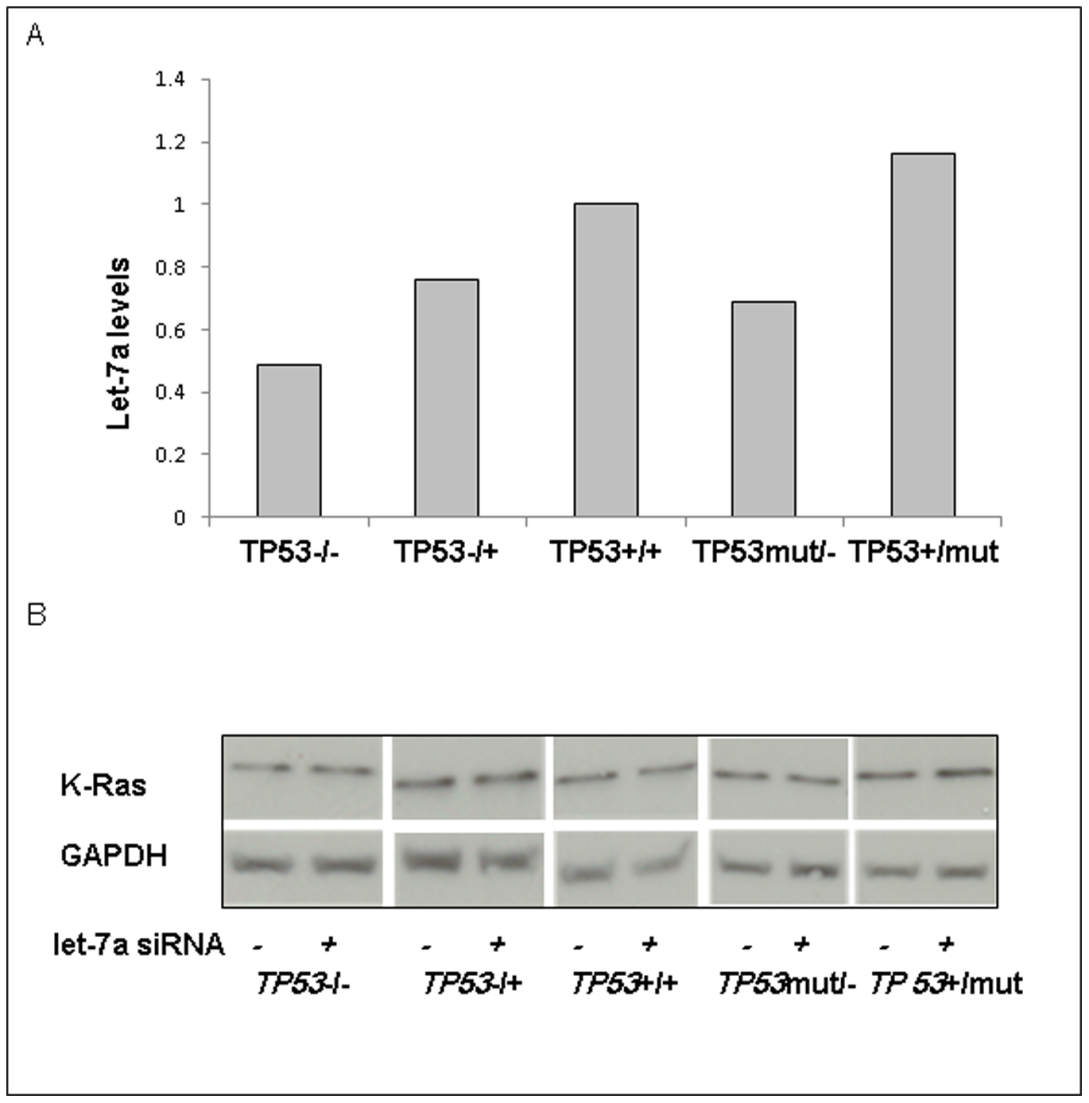
Let-7a is expressed in all cell lines and inhibition of let-7a does not affect K-Ras protein levels. A) Let-7a expression was measured via qRT-PCR. Data shown represents a single qRT-PCR performed in triplicate. B) Cells were transfected with 100 nM of let-7a inhibitor for 24 h. Protein for control and for let-7a inhibited cells were collected for immunoblotting for K-Ras expression. GAPDH served as a loading control.

Let-7a expression in HCT116 cell lines was measured by qPCR. Let-7a expression was detected in all cell lines (Figure 2A). There was no pattern of change in let-7a levels which corresponded to alterations in *TP53* genotype.

### Let-7a does not Regulate K-Ras Protein Levels

Let-7a gene expression was inhibited >90% by qPCR. K-Ras protein levels were assessed across the *TP53* genotypes in the presence or absence of let-7 inhibition. K-Ras protein levels were not altered by let-7a inhibition (Figure 2B).

### Let-7a Negatively Regulates K-Ras Activity

**Figure 3:**
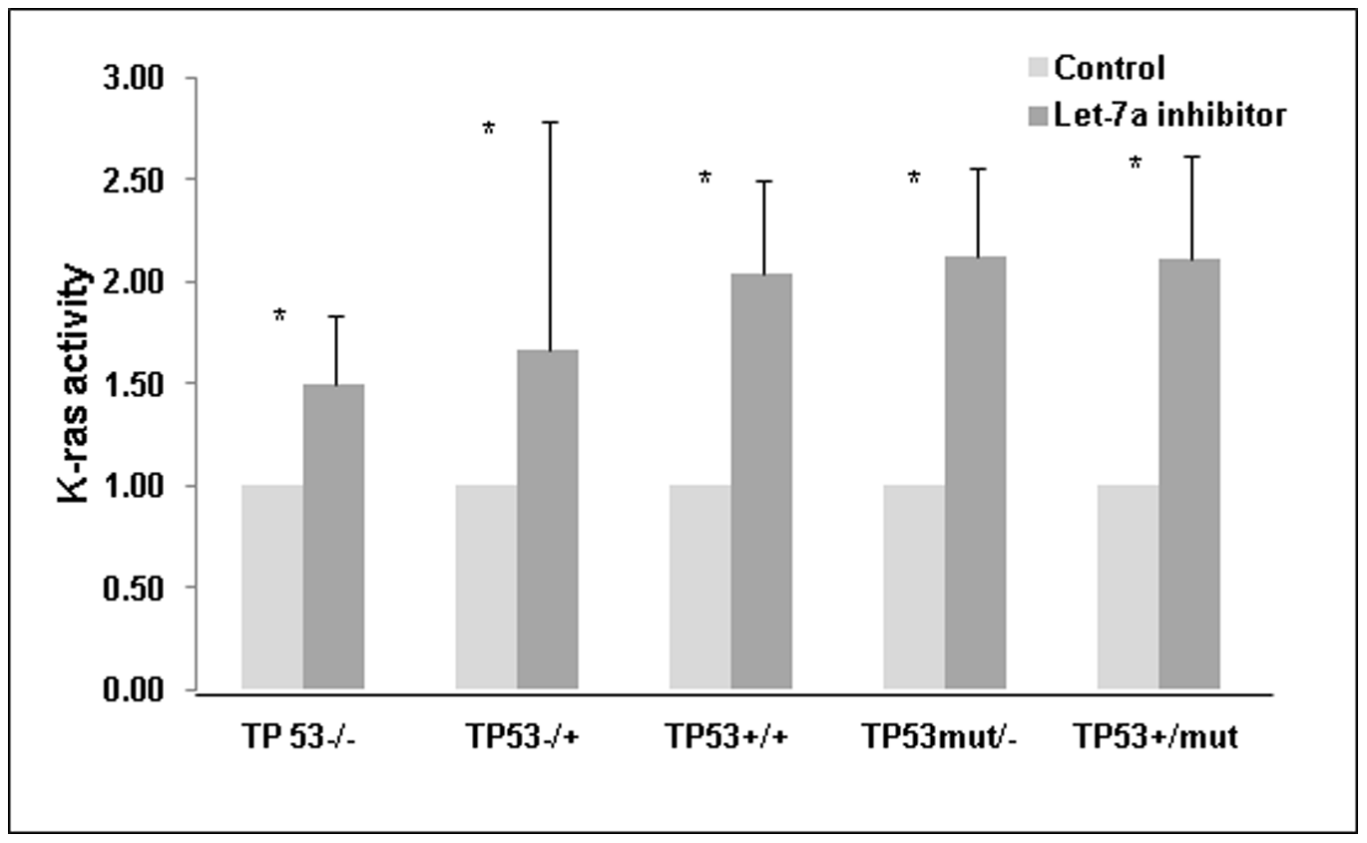
K-Ras activity is increased in cells with let-7a inhibition. Cell lysates were collected following 72 h incubation and subjected to a Raf-pull down assay which measures K-Ras activity. Data shown represents three experiments done in triplicate. Error bars show SD. *p<0.05.

The effect of let-7a inhibition on K-Ras activity was also determined. With let-7a inhibition, K-Ras activity increased across all *TP53* genotypes (Figure 3). We found that K-Ras activity increased by 50% to 112% compared to let-7a expressing cells (p<0.05). The percent increase in K-Ras activity across all cell lines was not clearly associated with *TP53* genotype and whether knockout or mutant alleles were present.

### Cells with Wild-type *TP53* Alleles are Sensitive to CRT and let-7a Inhibition Decreases Response to CRT

**Figure 4:**
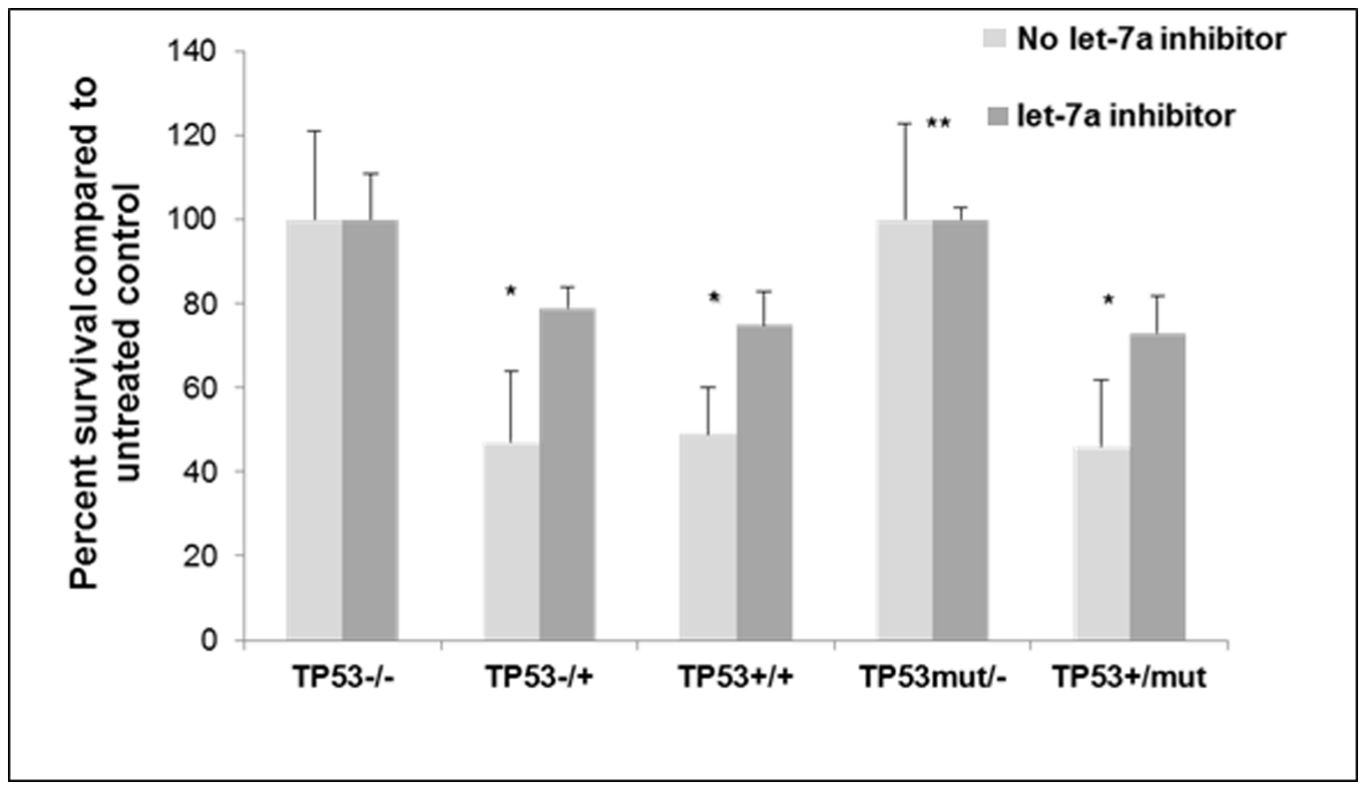
After let-7a inhibition, cell viability is partially rescued in CRT-sensitive cells. Cells with varying *TP53* status +/− let-7a inhibitor were treated with 5-FU followed by radiation. Cell viability was measured. Data shown represent the average viability as a percent of untreated cells. *p<0.05, **p>0.05.

Cells harboring different *TP53* genotypes were treated with CRT and cell viability was measured (Figure 4). Cells lacking any wild-type *TP53* allele were resistant to CRT, whereas cells harboring a single wild-type *TP53* allele exhibited approximately 50% cell death. CRT treatment was performed again with let-7a inhibition, which decreased CRT-induced cell death by nearly 20% in cells harboring at least one wild-type *TP53* allele (p<0.05). Cells with homozygous loss of wild-type *TP53* alleles (^−/−^ and ^mut/−^), which correlated with highest K-Ras activity (see Figure 1B), exhibited no change in cell viability regardless of changes in let-7a expression levels.

## Discussion

*KRAS* and *TP53* are common somatic mutations with predictive and prognostic value in patients with CRC. The detection of select *KRAS* mutations has been associated with increased risk of disease relapse and death [23] and has predicted resistance to anti-EGFR therapies in CRC [9]. Similarly, select *TP53* alterations have been associated with poor outcomes in CRC [8]. We have also reported that the concurrent detection of *TP53* and *KRAS* mutations in patients receiving neoadjuvant CRT for rectal cancer predicted failure of pathologic complete response [10]. In this investigation, we sought to better understand a possible interaction between the two genes. We identified let-7a as a potential link between *TP53* and *KRAS*. Prior characterization of let-7a has revealed its role in controlling cell cycle progression and division in human lung and colon cancers [24], [25]. Moreover, Johnson et al. [15] discovered that let-7 negatively controlled *KRAS* expression in lung cancer. In addition, Ruzzo et al. [17] reported an improved overall survival in patients with elevated let-7a within a *KRAS* mutant colorectal cancer population treated with anti-EGFR therapy, suggesting a tumor inhibitory role for let-7a in *KRAS*-activated tumors. *TP53* has been known to transcriptionally regulate the expression of miRNAs [26], [27]. Therefore, we theorized the existence of a series of regulatory steps from *TP53* to let-7a to *KRAS*. We discovered that oncogenic K-Ras activity was regulated by *TP53* genotype and let-7a; and changes in both influenced response to CRT.

In our study we utilized CRC cells harboring various *TP53* genotypes. These cells were derived from a parental cell line harboring the gain of function *KRAS* mutation located on codon 13 [28], [29]. There is rationale for utilizing cells with knockout and mutant *TP53* alleles in our study. Despite the common loss of wild-type *TP53* alleles, the knockout and mutant *TP53* alleles are not alike. Complete loss of *TP53* alleles results in corresponding loss of tumor suppressor activity while mutant *TP53* alleles may express p53 proteins that lose tumor suppressor activity but also gain oncogenic properties [21]. However, in this investigation we were unable to find consistent differences between KO and mutant *TP53* alleles with regards to K-Ras activity at baseline and in response to let-7a inhibition, nor in response to CRT.

As noted previously, we observed changes in K-Ras activity with inhibition of let-7a expression. However, we did not observe corresponding changes in K-Ras protein expression, which conflicts with prior reports demonstrating let-7 miRNA regulation of K-Ras expression [30], [31]. Nevertheless, our findings are consistent with established regulatory mechanisms of miRNA. Indeed, miRNA can regulate genes through a variety of mechanisms from mRNA translation to stability in the cytoplasm to cell cycle activity [14], [24]. The differences between our results and those of prior reports could also be due to differences between species or cell lines utilized. Thus, while K-Ras expression remained unchanged by let-7a inhibition, our experiments showed that K-Ras activity was affected by let-7a. In addition, cells harboring homozygous wild-type *TP53* alleles expressed the lowest K-Ras activity, which suggests that wild-type *TP53* alleles may suppress K-Ras activity. Furthermore, our results are consistent with a report in pancreatic cancer, showing an increase in K-Ras activity when mutant *KRAS* was paired with mutant *TP53* [32]. Finally, K-Ras activity clearly increased when let-7a was inhibited regardless of *TP53* genotype, further suggesting let-7a regulation of K-Ras activity.

Chemotherapy and radiation are important components of the multimodal management of surgical patients with rectal cancer; and neoadjuvant CRT has been associated with downstaging of disease and decreased local recurrence [33], [34]. In our investigation, we discovered that cells lacking wild-type *TP53* alleles were resistant to CRT and exhibited no cell death when treated with CRT. In contrast, cells with at least one wild-type allele (*TP53^+/+^, TP53 ^mut/+^*, and *TP53* ^−/+^) had approximately 50% cell death when treated with CRT. These results are consistent with the results from our prior clinical trials wherein patients with double mutations (*KRAS* and *TP53*) failed to demonstrate a pathologic complete response after treatment with neoadjuvant CRT [10]. When let-7a expression was inhibited (leading to increased K-Ras activity), cells lacking wild-type *TP53* alleles (*TP53^−/−^*and *TP53^mut/−^*) had 100% cell survival. However, in cells harboring at least one wild-type *TP53* allele (previously showing some cell death), cell survival increased in response to let-7a inhibition. These results have two important implications: (1) *TP53* genotype influences K-Ras activity and may predict response to CRT and (2) let-7a expression levels influence K-Ras activity and may alter response to CRT.

In summary, we provide insight into potential mechanisms linking *KRAS*, *TP53*, and let-7a. Although double mutants are relatively rare, the condition appears to have a poor phenotype with resistance to CRT. Our results contribute to a better understanding of the connection between *KRAS* and *TP53*. Knowledge of the pathways linking *KRAS*, *TP53*, and let-7a may provide greater insight into the mechanisms driving the poor phenotype observed in certain mutations in CRC. While we acknowledge that the limitations of our study include the lack of *in* *vivo* modeling and the limitations of one cell line, our focus was on the investigation of the potential cross-talk between let-7a and p53, which we clearly demonstrate in our model. It is feasible that this interaction may be cell line specific or may depend on other aberrant pathways in HCT-116 (MSI-H, *PIK3* mutant). Future work will focus on investigating the potential mechanisms of interaction between let-7a and p53 across different colorectal cell lines and with different molecular profiles.
